# We (Still!) Need to Talk About Valence: Contemporary Issues and Recommendations for Affective Science

**DOI:** 10.1007/s42761-023-00217-x

**Published:** 2023-09-02

**Authors:** Eric A. Walle, Daniel Dukes

**Affiliations:** 1 University of California, Merced, Merced, USA; 2 University of Fribourg, Fribourg, Switzerland

**Keywords:** Emotion valence, Discrete emotions, Functionalism, Emotion measurement

## Abstract

Valence is central to the experience of emotion. However, to the detriment of affective science, it is often ill-defined and poorly operationalized. Being more precise about what is meant by valence would make for more readily comparable emotion stimuli, methodologies, and results, and would promote consideration of the diversity, complexity, and function of discrete emotions. This brief review uses prior literature and an informal survey of affective scientists to illustrate disagreements in conceptualizing valence. Next, we describe issues of valence in affective science, particularly as they pertain to the emotion process, the functions of emotion, and precision in empirical research. We conclude by providing recommendations for the future of valence in affective science.

Affective scientists generally concur that valence is an essential aspect of emotion (e.g., Barrett, 2006; Dukes et al., 2021). However, there is also pervasive disagreement in how researchers conceptualize this basic construct. This brief review highlights significant inconsistencies in how valence is defined and operationalized by contemporary emotion researchers. Moreover, we argue that many conceptualizations of valence risk: (1) oversimplifying emotional experiences, (2) neglecting the functions of emotions, and (3) imprecisely measuring emotion. We conclude by suggesting two means for advancing theoretical and empirical approaches to the study of valence in affective science.

## Valence: An Unfocused and Ill-defined Construct

Given the prominence of valence in theories of emotion, it is perhaps surprising that so much confusion exists about how it is defined and operationalized. Indeed, many contemporary researchers of affective science have highlighted this issue (see Charland, 2005a, b; Higgins, 2016; Teroni, 2018). In her excellent review, Colombetti (2005) voiced several important criticisms. She points out that numerous phenomena can be identified as valenced (i.e., possessing positive and negative qualities), such as behaviors, goals, hedonics, and morality/norms. These aspects are not only distinct from one another, they can also be competing. For example, schadenfreude may feel good (positive), but may also be morally wrong (negative). Likewise, avoiding people may be adaptive (positive) when fearful of pathogens, but may also be goal incongruent (negative) if you want to throw a child’s birthday party. Unfortunately, discrete emotions are often categorized as being either positive or negative, even when research indicates such distinctions are less well-defined (An et al., 2017).

**Fig. 1:**
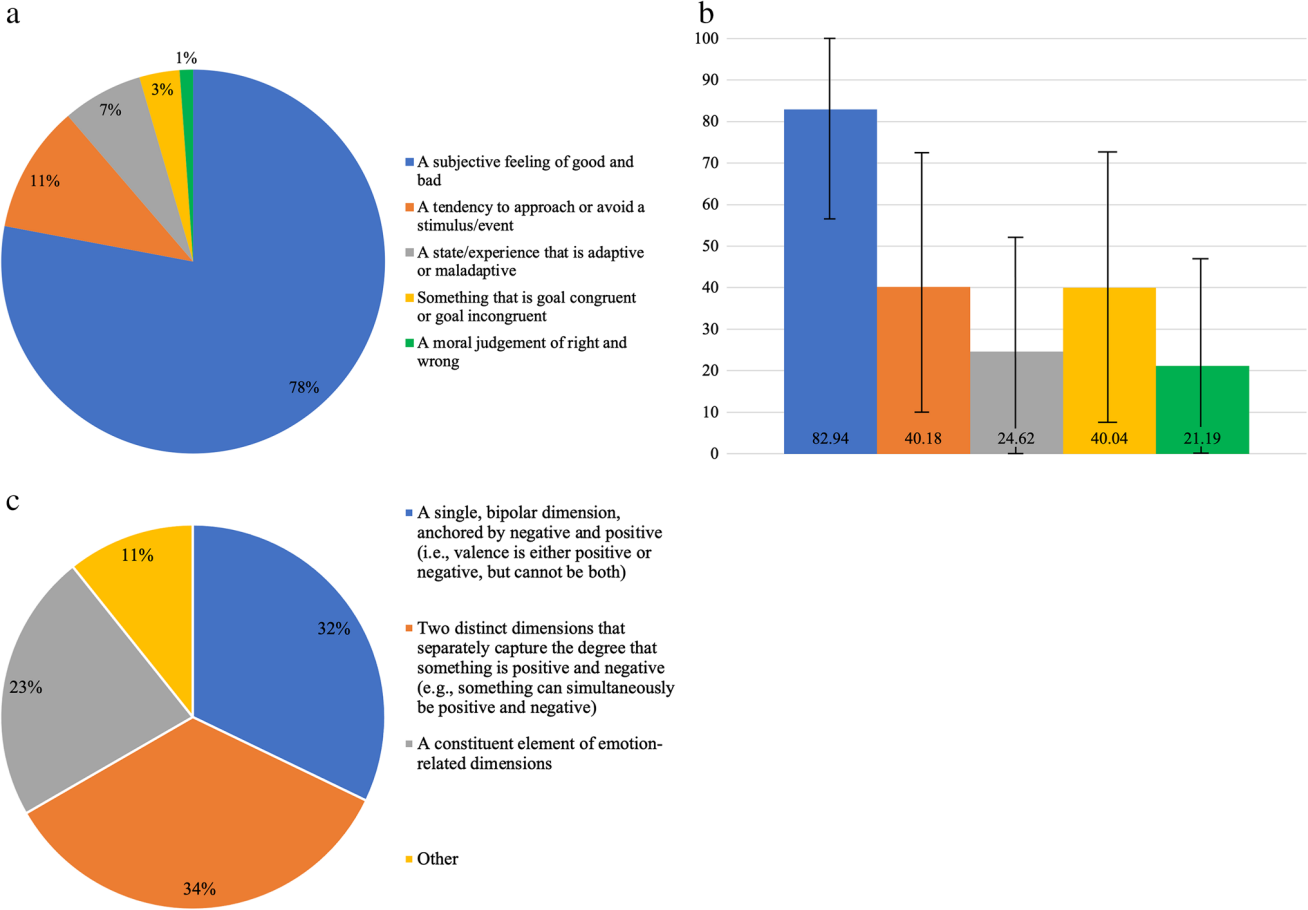
Informal survey of affective scientists. (**a**) which description best characterizes your conceptualization of valence?, (**b**)Please rate (100 = totally agree) the extent to which you agree with the following characterizations of valence, (**c**) In my view, valence is: *Note.* Anonymous survey data collected using Qualtrics. Respondents (*N* = 175) were recruited by contacting members of the editorial boards of the journals  *Emotion, Emotion Review,* and the *Journal of Emotion and Psychopathology*, and listservs maintained by the European Society for Cognitive and Affective Neuroscience, Human Affectome Project, International Society for Research on Emotion, Society for Affective Science, Swiss Center for Affective Science, and a general mailing list of philosophers "Philos-L.". Error bars represent +/-1 *SD*

Our own informal survey of affective scientists indicates the extent of this confusion (see Fig. 1). When asked to choose between five different characterizations of valence taken from the literature, 79% of the participants chose “a subjective feeling of good or bad” (see Fig. 1a). However, this consensus masks underlying ambiguity. Specifically, when asked to rate (0–100) the extent to which valence was characterized by the previous options, the same participants also characterized valence as “something that is goal congruent/ incongruent” (*M* = 40.04) and as “a tendency to approach or avoid a stimulus or event” (*M* = 40.18), suggesting there are multiple characterizations of valence endorsed by researchers (see Fig. 1b). Moreover, the variability within these ratings further demonstrates the diversity in how researchers conceptualize valence.

Strikingly, there is even disagreement about the structural properties of valence (see also Colombetti, 2005; Soloman, 2001). Consider the dimensionality of valence. Valence/pleasantness has not only been considered a bipolar dimension (e.g., Russell & Barrett, 1999), meaning an object’s valence must lie somewhere between extremely pleasant and extremely unpleasant, but also as two independent unipolar dimensions, meaning something can be simultaneously both pleasant and unpleasant (e.g., Cacioppo & Berntson, 1994; Watson & Tellegen, 1985). Debate on the dimensionality of valence extends to whether the construct is a distinct dimension at all. Researchers have suggested that valence is integrated within distinct appraisal dimensions (see Scherer, 2013), inseparable from the appraisal dimensions themselves, rather than an independent polarized dimension (e.g., Davitz, 1969). Such disagreement was similarly apparent in our survey data (see Fig. 1c).

Valence undoubtedly plays an important role in the emotion process. However, the above discrepancies in conceptualizing this construct have contributed to issues in affective science. Below we describe three such concerns and highlight empirical research that better considers valence.

## Issues of Valence in Contemporary Affective Science

### Oversimplification of Emotion

Research incorporating valence can neglect the qualitative distinctions of discrete emotions. Specifically, researchers often categorize “negative” emotions and “positive” emotions into two groups or select a single negative and a single positive emotion to represent all emotions in their respective categories. Lerner and Keltner (2000) point out how, in decision making research, emphasizing valence can oversimplify discrete emotions, such as how fear and anger differentially influence risk perception. Likewise, research on emotional development typically manipulates or measures positive or negative affect or approach and avoidance behavior (e.g., joy vs. fear to elicit approach or avoidance of a toy), but not discrete emotions (see Walle & Campos, 2012). Research experimentally manipulating discrete emotions illustrates meaningful differences beyond categorizations of emotion valence (e.g., Lench et al., 2011; Roseman et al., 1994; Walle et al., 2017).

Elsewhere, utilizing valence as a general term in clinical psychology can obscure the specific quality of individuals’ emotional experiences. For example, perceiving that a terminally ill patient feels “negatively” may lead a clinician to conclude the patient is depressed about the prognosis, thus missing the anger and guilt experienced by the individual reflecting on their past life choices (see Main et al., 2017). Research considering the downstream consequences of emotions demonstrates the complexity a priori definitions of valence. For example, discrete emotions typically labeled “negative”, such as anger and fear, can promote positive behavioral change and collective action in the name of progress (e.g., Tannenbaum et al., 2015; Wlodarczyk et al., 2017), and wives’ expressions of anger and contempt (but not sadness) during spousal conflict discussions correspond with improved martial satisfaction (Gottman & Krokoff, 1989). In this way, valence viewed as an essential element of the emotion process, rather than constituting the emotion proper, can clarify the important ebbs and flows of affective experiences.

### Neglecting the Functions of Emotion

Categorizing discrete emotions as positive or negative neglects the complex ways in which organisms relate to their environment—the crux of emotion (Campos et al., 1994). For example, while experiencing guilt and shame is typically reported as negative, these emotions can motivate pro-social, positive reparative behaviors, and thus re-acceptance (Keltner & Haidt, 1999). Moreover, an absence of appropriate guilt and shame can result in social stigmatization and be a sign of psychopathology (e.g., Gramzow & Tangney, 1992; Muris et al., 2016). Similarly, characterizing emotion-related behaviors of valence as approach/avoidance overlooks the equifinality of emotion (Campos et al., 2004): fear can prompt one to fight (approach), flee (avoid), or freeze (neither).

When these different and competing characterizations are considered, distinguishing emotions as “positively” or “negatively” valenced begs the question: in what way? The more apposite issue relates to an emotion’s function. Research on emotion regulation and wellbeing provide appropriate illustrations. For example, adults, adolescents, and even children utilize discrete emotions to achieve their goals, such as expressing happiness during collaboration, and anger, but not sadness, during confrontation (e.g.,López-Pérez et al., 2023; Tamir & Ford, 2012). Likewise, researchers of positive psychology have emphasized the functions of positive emotions (e.g., Shiota et al., 2014), with discrete emotions serving a positive (adaptive) function distinct from their typically designated valence. For example, while positive emotions were generally thought to increase susceptibility to the persuasiveness of an argument (e.g., Bless et al., 1990), recent research demonstrates that the effect depends on which “positive” emotion was manipulated (e.g., Griskevicius et al., 2010). Thus, considering the function of discrete emotions rather than their a priori valence can reveal important differences in how they operate in interpersonal contexts.

### Empirical Imprecision

The instrumentalization of valence can lead to imprecision in emotion-related stimuli and measures. Consider a commonly used set of stimuli, the International Affective Picture System (IAPS; Lang et al., 1998). Based on the assumption that “all emotions can be classified in two-dimensional space, as coordinates of affective valence and arousal” (Lang, 1995, p. 372), stimuli include ratings for their elicitation of negative or positive feelings (1- to 9-point scale). These stimuli have proved useful as affective science developed, but recent findings of context-dependency of emotion perception (e.g., Hassin et al., 2013; Reschke & Walle, 2021) demonstrates the need to move beyond context-free, static pictures. For example, how does one compare images of a dead body pulled through the street (image 9252, valence = 1.98) with a vomit-filled toilet (image 9325, valence = 1.89), or an erotic couple (image 4660, valence = 7.40)? The valence rating is minimally informative in assessing the quality (e.g., sadness, disgust, fear, jealousy) of the emotional experience. Moreover, the emotion elicited depends on their relational significance to the observer. Perhaps the dead body was a ruthless dictator, and the toilet scene conjures a humorous memory of college, yet both are categorized as negative, whereas the image of the erotic couple, perhaps portraying victims of an internet privacy hack, is characterized as positive.

Measures assessing emotion by using valence can be similarly imprecise. For example, the Positive and Negative Affective Schedule (PANAS; Watson et al., 1988) is routinely used to assess individuals’ emotional state. But is it sensible to lump guilty, scared, and hostile into a singular “negative” experience? Or in assuming that excited is necessarily a “positive” experience? Thus, while this measure may have adequate internal consistency, concerns of ecological validity abound. Indeed, critical research of the PANAS indicates overlap in participant responses of negative and positive scales (Harmon-Jones et al., 2009), and that positive affect should be separated into discrete emotions (Egloff et al., 2003). Having highlighted numerous concerns regarding measures of valence or measuring just a single emotion to assess individuals’ emotional experiences, Harmon-Jones et al. (2016) created the Discrete Emotion Questionnaire. We view this new tool, while admittedly a “work in progress” according to the authors, a meaningful step toward more precise assessment of emotion experiences.

## The Future of Valence

Valence is a crucial component of affective experiences, producing “the heat of emotion” (Charland, 2005b). Thus, if affective scientists are to agree to disagree about the nature of valence, it is imperative they explicitly define and operationalize the construct in their research. Indeed, many affective scientists do define emotion, and their research is better for it. Defining valence would facilitate comparisons across studies and may encourage efforts within the field of finding a more unified conceptualization of what valence is (and what it is not; see Prinz, 2010). Thus, if valence is “a subjective feeling of good or bad” (see Fig. 1a), researchers would be pushed to operationalize “subjective feeling” in order to measure “good” or “bad.” Furthermore, studies adopting similar definitions of valence could be properly compared in metanalytic research, as well as contextualized with emotion theory, such as the appraisal theories of Roseman (1984) and Scherer (2013) that emphasize pleasantness (appetitive/aversive for Roseman) and goal congruency (motive consistency for Scherer) in conceptualizing valence (e.g., Frijda et al., 1989).

The above recommendation should be viewed as a minimum for the future of valence in affective science. Below we outline two further recommendations that can better situate valence as a productive construct in the future of affective science.

### Hone the Construct of Valence

Rather than agreeing to disagree on conceptualizing valence, research is needed to capture more precisely what it means for an emotional experience to feel good or bad; a particular state to feel better or worse. Given that existing measures in affective science inherently have some level of measurement error, factor analysis is one means to identify which and how many elements constitute valence. A fundamental consideration rests in determining the constituent aspects of valence. When we asked affective scientists in our survey to rate their agreement with 5 common characterizations of valence, a plurality (34%) rated all above a score of 10 (out of 100), with 71% of respondents rating at least 3 options above this value. Likewise, our survey found considerable disagreement in researchers’ views regarding the dimensionality of valence. This demonstrates that researchers generally agree that valence is (or may be) a multi-component construct. Perhaps this consensus of valence as multifaceted is valid, but not yet empirically confirmed.

Factor analysis could be utilized to identify distinct elements of valence.^1^ Appraisal researchers of emotion were early adoptees of factor analysis to test assumptions of appraisal theory (e.g., Smith & Ellsworth, 1985) and more recently this technique has been utilized to examine emotion regulation (Naragon-Gainey et al., 2017; Seligowski & Orcutt, 2015). Exploratory factor analysis could be utilized to consider and identify different aspects of the valence construct. For example, researchers could analyze participant responses of various components that may comprise valence (e.g., pleasantness, goal congruency, behaviors) to parse out distinct and overlapping elements. Once identified, subsequent confirmatory factor analysis would test the measurement model. Identifying and differentiating the component elements would propel affective science past idiosyncratic definitions of valence and toward a more agreed upon definition of valence. Additionally, methodological advances may facilitate further empirical testing of valence. For example, research in neuroscience has examined the multidimensionality of valence (e.g., Viinikainen et al., 2010) and it has even been suggested that valence differs in dimensionality at the micro and macrolevel (Shuman et al., 2013). We encourage such approaches in future affective science research to better understand valence.

### Un-valence Discrete Emotions

We advocate that valence be considered a component of each emotional experience, rather than inherent to any specific emotion. Indeed, Solomon and Stone (2002) argued that valence falsely dichotomizes emotions into positive and negative experiences when it would be more precise to separately describe aspects of an emotional episode by hedonic tone, morality, adaptability, and relation to goal congruency. Thus, valence is not inherent to a discrete emotion, but rather a constituent aspect of each emotional experience. For example, anger is neither positive nor negative (whether in, e.g., hedonic, behavioral, or moral terms), but can be either, or both. Importantly, this is not simply recognition that an emotion can be either positive or negative; valence is distinct from the emotion proper (see Charland, 2005a). Rather than research attempting to categorize emotions into positive or negative valence (e.g., Noordewier & Breugelmans, 2013), valence, clearly defined and operationalized, could be used to better understand nuance within emotional experiences—the sweet taste of revenge; the bittersweet memory; and the relief of confession. Much remains to be explored in affective science utilizing valence in such a fashion.

This shift may also increase consideration of a greater number of discrete emotions that struggle to map onto a dichotomized valence categorization of emotion. For example, if “righteous anger” and “revenge” can feel positive, perhaps they are not part of an anger family on the left side of a circumplex, and instead distinct emotional experiences not captured by existing delimitations. Indeed, as emotion research crosses cultural boundaries, it has become increasingly difficult to plot some emotions along a valence axis. Some interesting examples include amae (interpersonal acceptance; Kitayama & Markus, 1990), Fremdschämen (vicarious embarrassment; Melchers et al., 2015), gluckschmerz (pain at another’s fortune; Smith & van Dijk, 2018), and kilig (feeling butterflies in the stomach from romantic attraction; Lomas, 2016). Doing so would also bring into greater focus “neutral/non-negative” emotions (e.g., surprise, awe, and excitement) that heretofore have failed to fit neatly into the valence dichotomy, as well as the role of valence in emotion-related processes, such as empathy (Wondra & Ellsworth, 2015).

## Conclusion

Our brief review of the literature and corresponding informal survey demonstrates confusion surrounding valence in affective science. This lack of clarity risks leading researchers to oversimplify the emotion process, ignore the functions of emotion, and conduct imprecise research. Despite previous scholarship noting issues relating to valence (e.g., Charland, 2005a; Colombetti, 2005; Solomon, 2001), disagreements and confusion surrounding this construct persist. Given the prominence of valence in both theory and research, explaining what is meant when referencing valence seems an obligatory step for affective scientists, and still further progress is called for if valence is to meaningfully advance affective science.

## Footnotes

1 We wish to thank an anonymous reviewer for their productive feedback on this idea.

## Additional Information

### Data Availability

Data from the anonymous survey can be accessed here: https://osf.io/nxjeb/?view_only=e8640a8c83c44198bf45bc2f74fc4708.
